# Bioprospection of Selected Plant Secondary Metabolites as Modulators of the Proteolytic Activity of *Plasmodium falciparum* Plasmepsin V

**DOI:** 10.1155/2023/6229503

**Published:** 2023-06-21

**Authors:** Abdulhakeem Olarewaju Sulyman, Oluwapelumi Oluwaseun Aje, Emmanuel Oladipo Ajani, Rukayat Abiola Abdulsalam, Fatai Oladunni Balogun, Saheed Sabiu

**Affiliations:** ^1^Department of Biochemistry, Faculty of Pure and Applied Sciences, Kwara State University, P.M.B. 1530, Malete, Ilorin, Nigeria; ^2^Department of Biotechnology and Food Science, Faculty of Applied Sciences, Durban University of Technology, Durban, South Africa

## Abstract

Malaria is a devastating disease, and its management is only achieved through chemotherapy. However, resistance to available medication is still a challenge; therefore, there is an urgent need for the discovery and development of therapeutics with a novel mechanism of action to counter the resistance scourge consistent with the currently available antimalarials. Recently, plasmepsin V was validated as a therapeutic target for the treatment of malaria. The pepsin-like aspartic protease anchored in the endoplasmic reticulum is responsible for the trafficking of parasite-derived proteins to the erythrocytic surface of the host cells. In this study, a small library of compounds was preliminarily screened *in vitro* to identify novel modulators of *Plasmodium falciparum* plasmepsin V (*Pf*PMV). The results obtained revealed kaempferol, quercetin, and shikonin as possible *Pf*PMV inhibitors, and these compounds were subsequently probed for their inhibitory potentials using *in vitro* and *in silico* methods. Kaempferol and shikonin noncompetitively and competitively inhibited the specific activity of *Pf*PMV *in vitro* with IC_50_ values of 22.4 and 43.34 *μ*M, respectively, relative to 62.6 *μ*M obtained for pepstatin, a known aspartic protease inhibitor. Further insight into the structure-activity relationship of the compounds through a 100 ns molecular dynamic (MD) simulation showed that all the test compounds had a significant affinity for *Pf*PMV, with quercetin (-36.56 kcal/mol) being the most prominent metabolite displaying comparable activity to pepstatin (-35.72 kcal/mol). This observation was further supported by the compactness and flexibility of the resulting complexes where the compounds do not compromise the structural integrity of *Pf*PMV but rather stabilized and interacted with the active site amino acid residues critical to *Pf*PMV modulation. Considering the findings in this study, quercetin, kaempferol, and shikonin could be proposed as novel aspartic protease inhibitors worthy of further investigation in the treatment of malaria.

## 1. Introduction

Malaria is a potential life-threatening disease caused by the Plasmodium parasite, transmitted to humans through the bites of infected Anopheles mosquitoes [1]. The symptoms of malaria include fever, headache, chills, and flu-like illness and, if left untreated, can cause severe complications, including anemia, respiratory distress, cerebral malaria, and organ failure. There are several different species of Plasmodium (*P. ovale*, *P. malariae*, *P. knowlesi*, *P. vivax*, and *P. falciparum*) that can cause malaria in humans, with *Plasmodium falciparum* being the deadliest [2]. Malaria is most common in tropical and subtropical regions of the world, particularly in sub-Saharan Africa, though also occurring in parts of Asia, South America, and the Middle East.

The continued rise in the infection has led to a corresponding increase in the mortality rate of the infected individuals. This fatality was corroborated by the latest World Malaria reports: an increase of 241 million reported cases in 2020 compared to 227 million recorded in 2019 [3]. Similarly in 2020, the death estimate was pegged at 627,000; this was coming from the 11% (69,000) increase over the previous year [4]. However, it is disheartening to note that the African region [5] continues to carry a disproportionately high share of the global malaria burden [6]. In fact, in 2020, the region was home to 95% of all malaria incidences and 96% of deaths [7]. Sadly, children under 5 years of age accounted for about 80% of these deaths [8]; this may not be surprising perhaps due to their vulnerability as hinted by global health bodies like WHO and UNICEF. Malaria has been adjudged as the third most deadly cause of mortality for children below 5 years, indicating that in every 2 minutes, a child below 5 years dies of malaria [9]. Additionally, in 2021 alone, four African countries including Nigeria (31.9%), the Democratic Republic of the Congo (13.2%), the United Republic of Tanzania (4.1%), and Mozambique (3.8%) accounted for just over half of all malaria deaths worldwide [5]. The treatment of malaria depends on the severity of the disease, the species of Plasmodium causing the infection, and the individual's health status. The World Health Organization (WHO) recommends artemisinin-based combination therapies (ACTs) as the first-line treatment for uncomplicated malaria caused by P. falciparum. These medications typically include an artemisinin derivative, such as artemether or artesunate, and a longer-acting partner drug, such as lumefantrine or mefloquine. For severe malaria or cases caused by other species of Plasmodium, intravenous (IV) antimalarial drugs, such as quinine or artesunate, are often used. In some cases, a combination of IV and oral antimalarial drugs may be used. Additionally, supportive care, such as fluid replacement and treatment of complications, may be necessary.

Despite the aforementioned, vaccines have also been developed as additional therapeutic in the management of malaria. Typically, Mosquirix is a WHO-recommended vaccine for children against *P. falciparum* in Africa [5]. However, it is very expensive costing $20 (or more) for a full dose of 4 shots [10]. While antimalarial drugs have been effective in treating and preventing malaria, there are still several limitations associated with their use [11]. For example, one of the biggest challenges in the treatment of malaria is drug resistance. Resistance to antimalarial drugs has been reported in several regions around the world, particularly with regard to *P. falciparum.* This means that the parasites have developed the ability to survive and multiply in the presence of the drugs, rendering them less effective. Similarly, like all medications, antimalarial drugs can cause side effects, such as nausea, vomiting, dizziness, and headaches. More serious side effects, such as liver damage or low blood cell counts, can also occur with some medications. In addition, some antimalarial drugs require complex dosage regimens, such as taking multiple pills at specific intervals over several days. This can make it difficult for individuals to adhere to the treatment plan, potentially leading to incomplete treatment and increased risk of drug resistance. Furthermore, some antimalarial drugs are expensive and may not be readily available in low-income countries where malaria is most prevalent. Thus, making access to effective treatment difficult and thereby contributing to the ongoing transmission of malaria. Overall, while antimalarial drugs have been effective in reducing the burden of malaria worldwide, continued efforts are needed to develop new and improved drugs that are more effective, affordable, and accessible to those who need them most.

The life cycle of *P. falciparum* is comprehensive, with an asexual replication in human red blood cells (RBC), where most of the current drugs have their targets [12]. Following the invasion of RBC by merozoites, the parasite differentiates from the ring to the trophozoite and later to the schizont stage [13]. During the trophozoite stage, the parasite heavily relies on the uptake of nutrients from the host cells [14]. Upon invasion, *P. falciparum* degrades the host hemoglobin and changes the erythrocyte surface by exporting several proteins for the uptake and transport of nutrients [15]. With these modifications, the Plasmodium can rebuild and invade the host erythrocyte using exported proteins for its invasive growth and infection [16]. The exportation of proteins from the parasite to the host erythrocyte is done via a putative parasite-derived protein complex known as the translocon of exported proteins (PTEX), and this is found in the parasitophorous vacuole membrane (PVM) [17]. Studies on the much talked about exported protein in *P. falciparum* showed that they contain a pentameric localization motif known as *Plasmodium* export element (PEXEL) which constitutes a bifunctional export motif comprising a protease recognition sequence that is cleaved (by *Plasmodium falciparum* plasmepsin V), in the endoplasmic reticulum, from proteins destined for export, in a PEXEL arginine- and leucine-dependent manner. The malaria parasite, *Plasmodium falciparum*, produces a class of enzymes known as plasmepsins that degrade hemoglobin; the consequence is various symptoms presented by malaria patients [18]. Due to the essentiality of the exporting process to the erythrocyte membrane and the absence of homologues in the human host, PMV represents an excellent drug target. Despite the increasing amount of *Plasmodium falciparum* plasmepsin V (*Pf*PMV) inhibitors, there is still room for the discovery of novel chemical scaffolds with improved efficacy and reduced adverse effects. In the present study, PMV inhibition was sought by screening a small molecular weight library of natural compounds against PMV with emphasis on a thorough understanding of how the enzyme works towards developing probable inhibitors for drug discovery through advanced computational approaches and *in vitro* experimentation.

## 2. Materials and Methods

The chemical and reagents including casein, thioredoxin, chloramphenicol, kanamycin, isopropyl-D-thiogalactopyranoside (IPTG), Tris-HCl, NaCl, imidazole, Triton X, mercaptoethanol, HisTrap column, and glycerol used in this study were purchased from Sigma-Aldrich, United Kingdom. All other chemicals and solvents used were of analytical grade.

We extracted the PMV gene that spans the whole length (identified as PlasmoDB ID: PF3D7 1323500) from the genomic DNA of the 3D7 strain of *P. falciparum*. The process of cloning the *PfPMV* gene and creating plasmid DNA was performed at the Faculty of Life Sciences, School of Pharmacy and Biomolecular Sciences, Liverpool John Moores University, located in Liverpool, United Kingdom.

### 2.1. Overexpression and Purification of Plasmodium falciparum Plasmepsin V

The method described by Sambrook [19] was adopted for the cloning and purification of *Pf*PMV. The Trx-*Pf*PMVp37 was overexpressed in *E. coli* BL21 cells according to the method described by Boonyalai et al. [20]. Briefly, 10 ml of Luria-Bertani (LB) broth was transferred into a sterile 50 ml tube. Subsequently, 100 *μ*l of antibiotics (chloramphenicol) was added to the broth followed by 100 *μ*l BL21 cell from the stock. Thereafter, the cell was incubated at 37°C at 225 rpm until the cell density reached the midlog phase (OD_600_ of 0.636). Consequently, the tube containing the mixture was placed on ice for 5 minutes to stop the growth of the cells and centrifuged at 3000 rpm for 4 minutes. Thereafter, the supernatant from the centrifuged mixture was discarded, followed by the addition of 10 ml chilled CaCl_2_ to the resulting pellet, mixed gently, and then centrifuged again at 2000 rpm for 4 minutes. Again, the supernatant was discarded, and 400 *μ*l of chilled CaCl_2_ was added to the pellet and aspirated gently. Four (4) *μ*l of plasmid DNA (Trx-*Pf*PMVp37) was emptied into a 1.5 ml Eppendorf tube suspended on ice, and 100 *μ*l of the competent cell was introduced into the mixture, aspirated gently, and thereafter exposed to a heat shock (on heat block) for 5 minutes at 37°C. Following this, an equal volume of LB broth (100 *μ*l) was added to the mixture containing the cell and incubated for 90 minutes on ice. Two hundred (200) *μ*l from the incubated mixture was transferred into an LB broth containing chloramphenicol and kanamycin (100 *μ*l each), incubated overnight at 37°C at 225 rpm. To 5 ml of LB broth containing 50 *μ*l each of chloramphenicol and kanamycin in a tube was an addition of 100 *μ*l cultured cells, incubated for 120 minutes at 37°C with shaking at 225 rpm to each midlog phase (OD_600_ 0.4–0.8), and the absorbance of the culture containing Trx-*Pf*PMVp37 was measured after 120 minutes. Fifty (50) *μ*l of 100 mM IPTG following absorbance measurement was added to the medium containing Trx-*Pf*PMVp37, incubated for 3 h (for enzyme expression to occur), subsequently suspended on ice for 10 minutes to halt the reaction (growth), and later centrifuged at 10,000 rpm for 1 min. The supernatants were discarded while the pellet was kept in the freezer.

value="Exactly "400 *μ*l of bug buster reagents was added and vortexed gently. Exactly 1 ml of benzoanate was added to degrade all the nucleic acid that may be present (e.g., DNA and RNA). The medium was centrifuged at 16,000 × *g* for 20 minutes to pellet insoluble cell debris. The supernatant containing the soluble enzyme, plasmepsin V, was used immediately. The supernatant cells were suspended again in an extraction buffer (consisting of 50 mM Tris-HCl, pH 8, 0.5 M NaCl, and 5 mM imidazole) with additional supplements of 1.6 mM mercaptoethanol and 1% Triton X-100. The cell suspension was disrupted through sonication using the Sonics Vibra Cell VCX 750, and the fractions obtained were introduced into the histidine-tagged column connected to the HPLC system for purification. The protein that was attached was released in steps by using an elution buffer (containing 20 mM Tris-HCl, pH 8.0, 1 M NaCl, 5% glycerol, and 1 mM EDTA). The protein that was eluted was then concentrated using Vivaspin 6 with a 30 kDa MWCO (manufactured by GE Healthcare).

#### 2.1.1. Determination of Plasmepsin V Activity

To assess the proteolytic activity, casein was used as a substrate. A reaction mixture was prepared which consisted of 50 *μ*l of 2% (*w*/*v*) casein solution, 190 *μ*l of 50 mM glycine-HCl buffer (pH 3.4), and 10 *μ*l of the required concentration of the purified enzyme. The reaction mixture was then left to incubate at 35°C for 30 minutes, followed by the addition of 10% (*w*/*v*; 250 *μ*l) trichloroacetic acid (TCA) to terminate the reaction. Following this, the protein that had precipitated was eliminated through cold centrifugation, which was performed at 12,000 rpm for 10 minutes at a temperature of 4°C. The absorbance of the remaining liquid at 280 nm was then measured using a Hitachi U3000 spectrophotometer, which was manufactured in Tokyo, Japan. The definition of one unit (U) of enzyme activity was established as the quantity of enzyme required to increase the absorbance at 280 nm by 0.001 AU per minute under the specific conditions of the assay [21].

### 2.2. Screening of Natural Product Library

Initially, we tested the efficacy of 502 natural compounds obtained from Screen-Well® Natural Product Library (Enzo Life Sciences; https://www.enzolifesciences.com/BML-2865/screen-well-natural-product-library/) by subjecting them to screening. We established a standard of 50% activity inhibition at a 500 *μ*M concentration of the drug candidates. Based on this preliminary screening, we found six compounds which were later narrow down to three: kaempferol, quercetin, and shikonin (unpublished data).

### 2.3. Concentration-Dependent Inhibition of Plasmepsin V by Kaempferol, Quercetin, and Shikonin

The concentrations of kaempferol, quercetin, and shikonin were varied to determine the exact concentration(s) that will inhibit the activity of plasmepsin V. The reaction mixture contained 10 *μ*l plasmepsin V, 170 *μ*l 50 mM glycine-HCl buffer (pH 3.5), 10 *μ*l distilled water, 50 *μ*l 2% (*w*/*v*) casein solution, and 10 *μ*l of each compound at varied concentrations (125 *μ*M, 250 *μ*M, 500 *μ*M, 750 *μ*M, and 1000 *μ*M). Subsequently, the reaction mixture was incubated for 30 minutes at 35°C and quenched by the addition of 10% (*w*/*v*) TCA. The precipitated protein was removed by centrifugation at 12,000 rpm and 4°C for 10 minutes. The absorbance of the supernatant was measured at 280 nm [21].

### 2.4. Mode of Inhibition of Plasmepsin V and Determination of Kinetic Parameters

The mode of inhibition of plasmepsin V by kaempferol, quercetin, and shikonin with the highest % inhibition was carried out by varying concentrations of the substrate according to the modified method of Vishwanatha et al. [21]. The reaction mixture contained 10 *μ*l of plasmepsin V, 170 *μ*l 50 mM glycine-HCl buffer (pH 3.5), 10 *μ*l distilled water, 10 *μ*l test compounds (500 *μ*M and 750 *μ*M, respectively, of kaempferol and shikonin), and 50 *μ*l of varying concentrations (0.065 to 4 *μ*M) of casein. While the reaction mixture was incubated for 30 minutes at 35°C, it was followed by the inclusion of 10% (*w*/*v*) TCA. Thereafter, the precipitated protein was separated by centrifugation, performed at 12,000 rpm and 40°C for 10 minutes. The absorbance of the liquid that remained after centrifugation was measured at 280 nm using a Hitachi U3000 spectrophotometer (Tokyo, Japan). One unit (U) of enzyme activity was described as the quantity of enzyme needed to increase the absorbance at 280 nm by 0.001 AU per minute under the specific conditions of the assay [21]. The double reciprocal transformation was used to transform the data; the mode of inhibition and kinetic parameters were determined from the Lineweaver-Burk plot [22].

### 2.5. Computational Evaluation

#### 2.5.1. Molecular Docking

*(1) Protein and Ligand Acquisition, Molecular Docking, and Simulation*. The 3D crystal structure of *Pf*PMV (PDB ID: 4ZL4) was obtained from the Research Collaboratory for Structural Bioinformatics (RSCB) Protein Data Bank (https://www.rcsb.org; accessed January 15, 2023). The protein structure was prepared on the University of California San Francisco (UCSF) Chimera version 1.14 by removing water molecules, nonstandard naming, and protein residue connectivity [23]. The *x*-*y*-*z* coordinates of 4ZL4 active sites were defined using Discovery Studio version 21.1.0, as previously reported [24]. Before molecular docking, the protein structure was corrected by adding missing atoms in its side chain.

On the other hand, the reference drug, pepstatin, and the investigated compounds (kaempferol (ID: 5280863), quercetin (ID: 5280343), shikonin (ID: 479503), 5-hydroxy-2-methoxy-1,4-naphthoquinone (ID: 10104346), escin (ID: 16211024), and doramectin (ID: 9832750)) were accessed and downloaded from PubChem database (https://pubchem.ncbi.nlm.nih.gov/; accessed January 15, 2023). The compounds were then optimized using the Gasteiger charges within the Open Babel program plug-in on PyRx [25]. Thereafter, docking of the prepared protein (4ZL4) and optimized compounds (ligands) was performed using the AutoDock tool as previously described [25]. The docked complexes of the top three compounds were retrieved in PDB format for further molecular dynamic (MD) simulation.

The MD simulation was performed as previously reported [26] using the AMBER 18 package over a 100 ns period with the adoption of the FF18SB variant of the AMBER force field to describe the operating systems. Similarly, the ANTECHAMBER was used to generate the atomic partial charges of the ligands by utilizing general AMBER force field (GAFF) measures and restrained electrostatic potential (RESP). The Leap module's hydrogen atoms and Na^+^ and Cl^−^ counter ions were used to neutralize the systems. The amino acid residues were appropriately numbered, and the system was suspended inside an orthorhombic box of TIP3P water molecules in such a way that all atoms were within 8 of any box edge. The SHAKE algorithm was used in each simulation to constrict hydrogen atom bonds [27]. The CPPTRAJ module [28] was used for the postdynamic analysis of root mean square fluctuation (RMSF), root mean square deviation (RMSD), radius of gyration (RoG), and solvent-accessible surface area (SASA), and the resulting plots were generated in Origin V18 [29]. The Molecular Mechanics/GB Surface Area (MM/GBSA) method was used in estimating the binding free energy by averaging over the 100,000 snapshots taken from a 100 ns MD simulation trajectory [30].

### 2.6. Statistical Analysis

Except otherwise stated, all the enzyme assays were carried out in triplicate, and the results are expressed as mean ± standard error of the mean (SEM). The analysis was performed using one-way analysis of variance (ANOVA) and complemented with the Duncan multiple range tests. Differences were taken to be significant (statistically) at a 5% level of confidence (*p* < 0.05). GraphPad Prism 9.2.0 (GraphPad, La Jolla, CA, USA) and Origin data analysis package version 18.0 were used to plot all the graphs for the *in vitro* and *in silico* analyses [29].

## 3. Results

### 3.1. Screening of Small Molecular Weight Library of Compounds

**Figure 1:**
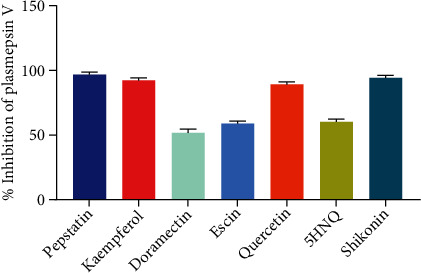
Screening of small molecular weight library of compounds expressed as percentage inhibition. Values are expressed as means ± standard error of the mean (SEM) of triplicate determinations. Differences were taken to be significant (statistically) at a 5% level of confidence (*p* < 0.05).

The data garnered on the inhibitory potential of the tested compounds at 1000 *μ*M against *Pf*PMV was in the order of shikonin (94%) > kaempferol (92%) > quercetin (89%) > 5HNQ (60%) > escin (59%) > doramectin (52%) as against 97% obtained for pepstatin (Figure 1). The analysis afforded the best three compounds/hits (shikonin, kaempferol, and quercetin) that were subjected to the dose-dependent evaluation against *Pf*PMV.

### 3.2. Inhibition of PfPMV Activity by Kaempferol, Quercetin, and Shikonin

**Figure 2:**
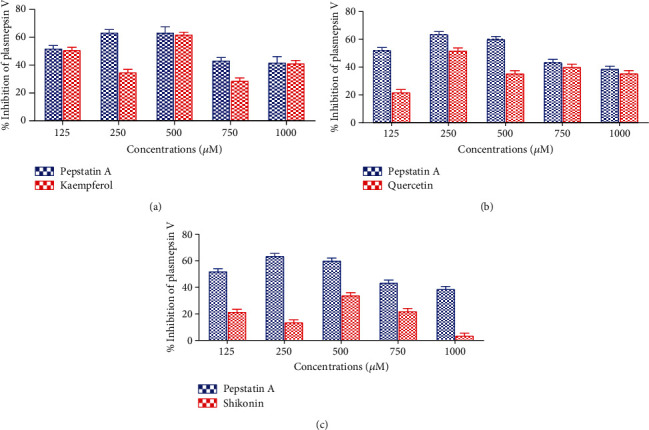
Effect of varying concentrations of (a) kaempferol, (b) quercetin, and (c) shikonin on plasmepsin V activity. Values are expressed as means ± standard error of the mean (SEM) of triplicate determinations. Differences were taken to be significant (statistically) at a 5% level of confidence (*p* < 0.05).

The result of *in vitro* inhibition of *Pf*PMV at varying concentrations of the compounds is presented in Figure 2. Kaempferol inhibited the proteolytic activity of *Pf*PMV with a significant (*p* < 0.05) effect exhibited at 500 *μ*M (Figure 2(a)) comparable to the standard, pepstatin A. While there was an inconsistent trend in the concentration-activity relationship between the samples (kaempferol and pepstatin A), however, the activity of kaempferol was stronger against PMV judging by the lowest half-maximal inhibitory concentration (IC_50_) value (22.4 *μ*M) when compared with pepstatin A (62.6 *μ*M) (Table 1). Similarly, Figure 2(b) reveals the best inhibition of quercetin on the activity of *Pf*PMV at 250 *μ*M though lower than pepstatin A. However, this observation in % inhibition was not correlated with the IC_50_ results as quercetin gave a lower IC_50_ value of 53.14 *μ*M as opposed to the reference, aspartic protease inhibitor, pepstatin (62.6 *μ*M) (Table 1). The highest % inhibition of *Pf*PMV by shikonin was obtained at 500 *μ*M. Shikonin activity for all the concentrations was reduced compared with pepstatin A at all levels (Figure 2(c)). Above all, going by IC_50_ results, all the compounds revealed superior activities against *Pf*PMV compared to the standard, with kaempferol being the best (Table 1).

**Figure 3:**
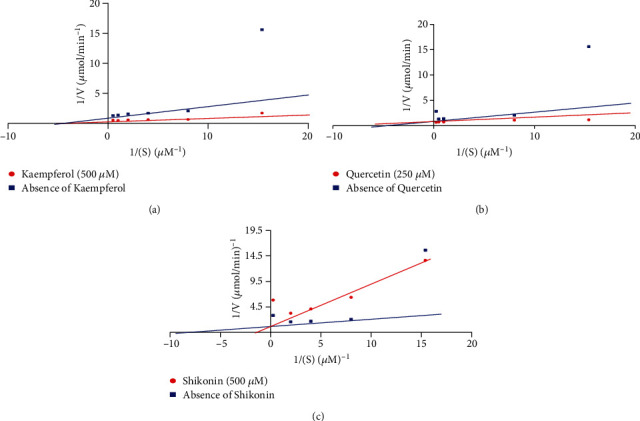
The double reciprocal plot of plasmepsin V in the presence and absence of (a) kaempferol, (b) quercetin, and (c) shikonin.

The result of the kinetics of inhibition of *Pf*PMV activity as depicted by the double reciprocal transformation plot revealed a decrease in the Vmax value (from 1.099 *μ*mol/min to 0.4110 *μ*mol/min) in the presence of kaempferol and a constant value for Km (0.0820 mM), suggesting a noncompetitive type of inhibition (Figure 3(a)). Additionally, a noncompetitive inhibition was similarly observed for quercetin with a constant Km value (0.079 mM) and a reduction in Vmax values between the control and the sample from 2.067 and 1.431 *μ*mol/min (Figure 3(b)) in the presence and absence of quercetin. The double reciprocal plot in the presence and absence of shikonin is shown in Figure 3(c) revealing an increase in the values of Km (from 0.073 *μ*M to 0.618 *μ*M) and a constant Vmax value of 1.262 *μ*mol/min suggestive of competitive inhibition.

### 3.3. In Silico Evaluation

The molecular docking results of the investigated metabolites against *Pf*PMV are shown in Table 2. The docking scores ranged between -5.3 and -8.7 kcal/mol, with kaempferol (-8.7 kcal/mol) having the highest negative score relative to pepstatin (-8.3 kcal/mol) (Table 2). The results of the binding energy components of the top three studied compounds (kaempferol, quercetin, and shikonin) and pepstatin with *Pf*PMV following MD simulation analysis over the 100 ns period are presented in Table 3. It was observed that quercetin had the highest negative binding energy (-36.56 kcal/mol) which was marginally or insignificantly (*p* > 0.05) higher than that observed with pepstatin (-35.72 kcal/mol) against *Pf*PMV (Table 3).

**Figure 4:**
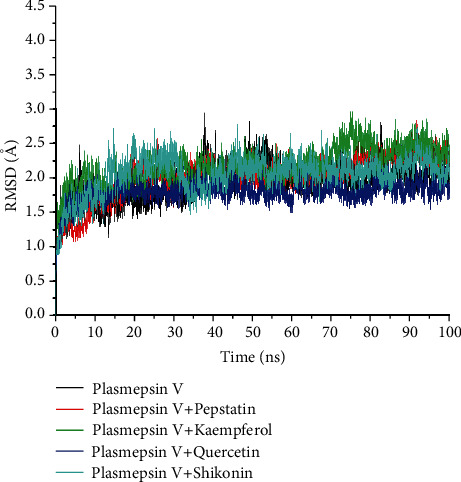
Comparative root mean square deviation (RMSD) profiles of alpha-C atoms of the plasmepsin V (*Pf*PMV) with plasmepsin V-pepstatin, plasmepsin V-kaempferol, plasmepsin V-quercetin, and plasmepsin V-shikonin systems over a 100 ns molecular dynamic simulation.

The overall average RMSD value of the unbound system (*Pf*PMV) was 1.92 Å which was significantly (*p* < 0.05) lower compared to all the bound systems including pepstatin-*Pf*PMV (standard) and the compounds except quercetin-*Pf*PMV complex (1.79 Å), although the significant increase of these values is within the acceptable limit of <3 Å (Table 4). The result here corroborated the findings from the thermodynamic energy profiles where quercetin had the highest negative binding energy value followed by the reference drug. The evaluation of the pattern of the RMSD plots revealed that convergence and divergence of all the investigated systems occurred around 15 and 35 ns, respectively (Figure 4), while there appear to be inconsistent patterns for the kaempferol and pepstatin systems towards 100 ns. However, shikonin and quercetin systems were relatively stable with the best stability observed with the latter.

**Figure 5:**
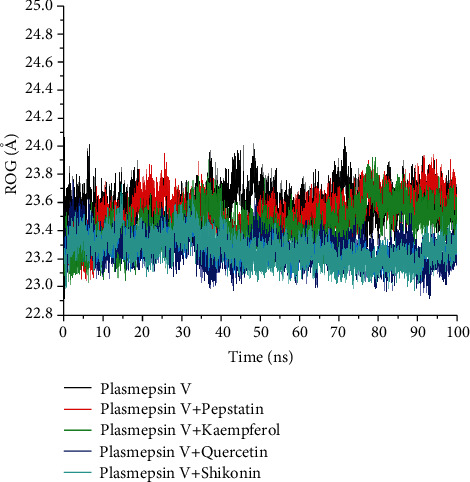
The radius of gyration (RoG) plots of protein backbone atoms of plasmepsin V (*Pf*PMV) with plasmepsin V-pepstatin, plasmepsin V-kaempferol, plasmepsin V-quercetin, and plasmepsin V-shikonin systems over a 100 ns molecular dynamic simulation period.

Contrary to the result from the RMSD for the studied compounds and reference drug, the mean RoG value of the apoenzyme (23.59 Å) was marginally higher compared to the bound systems (Table 4). The studied compounds most especially quercetin-*Pf*PMV and shikonin-*Pf*PMV complexes revealed the lowest RoG values (23.27 Å) as compared to the reference drug (23.53 Å) (Table 4). The least RoG values of quercetin and shikonin-bound systems buttress the binding energy result, particularly for quercetin. The findings of the comparative RoG plots of *Pf*PMV and the bound systems over 100 ns MDS revealed that the apoenzyme fluctuated more compared to the investigated complexes (Figure 5). There was an observed convergence of the systems around 10 ns followed by the steadiness of stability for the systems (after 10 ns) and maintained through the simulation period, particularly for quercetin and shikonin.

**Figure 6:**
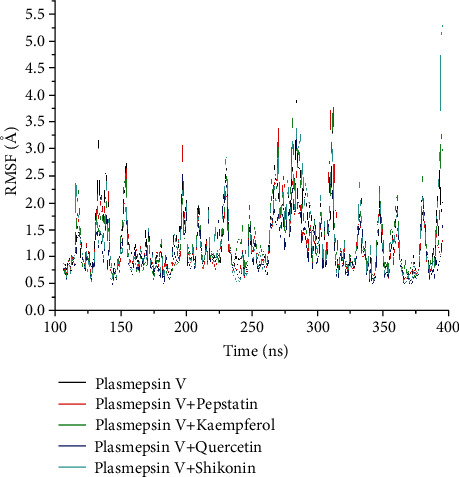
Comparative root mean square fluctuation (RMSF) plots of residue-based average C-*α* fluctuations of unbound plasmepsin V (*Pf*PMV) and bound complexes of plasmepsin V-pepstatin, plasmepsin V-kaempferol, plasmepsin V-quercetin, and plasmepsin V-shikonin observed over 100 ns.

The average RMSF value of the unbound was insignificantly (*p* > 0.05) higher (1.28 Å) compared to the bound systems except kaempferol-*Pf*PMV (1.29 Å) complex. The RMSF value of quercetin-*Pf*PMV system (1.09 Å) was observed to be the lowest of all systems, even better than the reference drug, pepstatin (1.23 Å) (Table 4). While the plots of the amino acid residues for *Pf*PMV and the bound systems are presented in Figure 6, kaempferol exhibited greater protein flexibility at residues 225, 250-270, and 280-300 compared to the unbound *Pf*PMV (Table 4). Overall, quercetin has a lower fluctuation pattern compared to the reference drug and the unbound *Pf*PMV (Figure 6).

**Figure 7:**
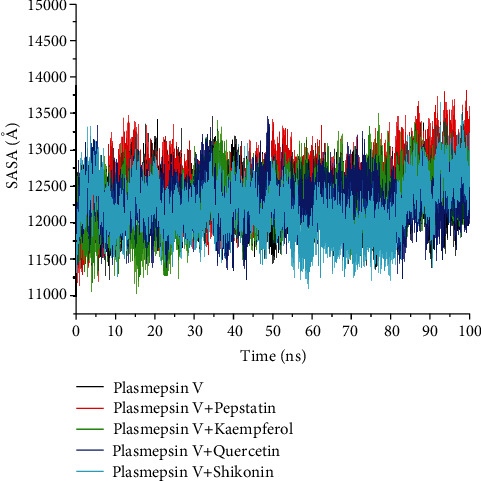
Comparative solvent-accessible surface area (SASA) plots of residue-based average C-*α* fluctuations of free plasmepsin V (*Pf*PMV) and plasmepsin V-pepstatin, plasmepsin V-kaempferol, plasmepsin V-quercetin, and plasmepsin V-shikonin systems over 100 ns MD simulation.

In a similar trend as RoG, the average SASA values of the top three investigated compounds, i.e., kaempferol (12297.89 Å), quercetin (12275.66 Å), and shikonin (12190.77 Å), were lower compared to the reference compound (12518.79 Å) and the unbound protein (12323.14 Å) (Table 4 and Figure 7) though the SASA value of pepstatin-*Pf*PMV complex was higher (*p* > 0.05) relative to the apoenzyme. Overall, shikonin was observed to have the least average SASA value; this was corroborated by the stability throughout most of the simulation period, up to 85 ns, whereas other systems revealed inconsistent fluctuations up until 100 ns.

**Figure 8:**
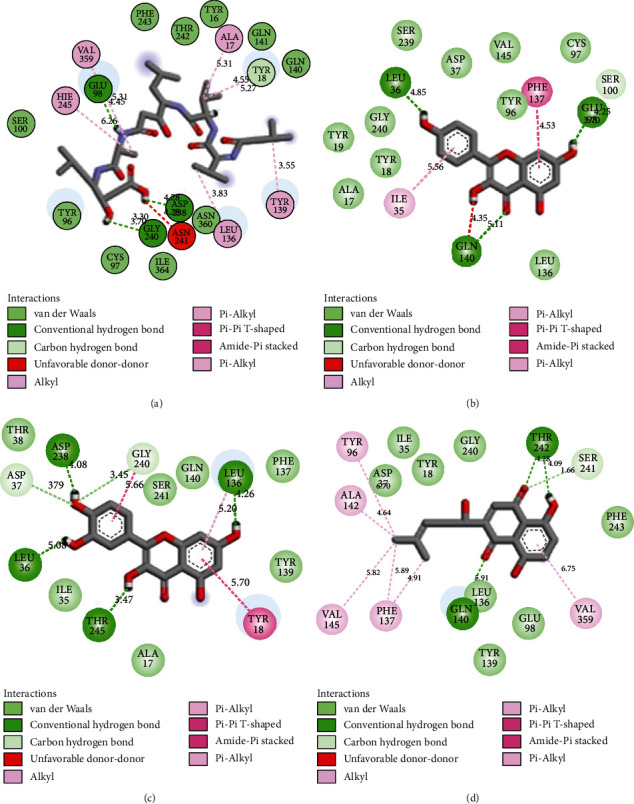
Interaction plots and types of (a) pepstatin, (b) kaempferol, (c) quercetin, and (d) shikonin with the active site amino acid residues of *Pf*PMV post-100 ns MD simulation.

The post-MD simulation interactions between the residues at the active site of *Pf*PMV with the investigated compounds are presented in Figures 8(a)–8(d). While most of the interactions are conventional H bonds, van der Waal forces, C-H bonds, and pi bonds, pepstatin revealed more interactions (20) comprising 10 van der Waals forces (Ser10, Tyr96, Cys97, Ile364, Asn36, Gln14, Gln14, Tyr16, Thr242, and Phe243), 3 convectional H bond (Asp238, Gly240, and Glu98), 1 C-H bond (Tyr18), 1 unfavourable donor-donor, 5 alkyls, and pi-alkyl (Figure 8(a)) compared to the studied compounds. Kaempferol-*Pf*PMV complex had 14 interactions consisting of 7 van der Waal forces (Thr38, Ser241, Gln148, Phe127, Tyr139, Ile35, and Ala17), 4 convention H bonds (Asp238, Leu136, Thr242, and Leu36) 2 C-H bonds (Asp37, Gly240), and 1 pi-pi T-shaped bond (Figure 8(b)) while quercetin (10 van der Waal forces (Ala17, Tyr18, Gly240, Tyr19, Ser239, Asp37, Val147, Cys97, Tyr19, and Leu136), 3 conventional H bonds (Leu36, Glu53, and Gln140), 1 C-H bond (Ser100), 1 pi-pi bond (Phe137), and 1 pi-alkyl (Ile35)) and shikonin (8 van der Waal forces (Asp37, Ile35, Tyr18, Gly240, Phe243, Glu98, Tyr139, and Leu136), 2 convectional hydrogen bonds (Thr242, Gln140), 1 C-H bond (Ser241), and 5 Alkyl and pi-alkyl (Val359, Phe137, Val145, Ala142, and Tyr96)) revealed 16 interactions each (Figures 8(c) and 8(d), respectively) for *Pf*PMV.

## 4. Discussion

The proteolytic enzymes present in malaria have both regulatory and effector functions in several crucial biological processes in essential pathogens such as *P. falciparum*; they have been identified as a potential antimalarial therapy [31]. Although efforts in the eradication of malaria have made great advancements in recent times; the latest concern was in the resistance of *P. falciparum* to all the available antimalarial drugs which have become a drawback in the fight against this scourge [32]. The discovery of new antimalarial therapies that will combat the challenges of drug resistance is therefore urgently required. *Plasmodium falciparum* plasmepsin V is an important aspartic protease found in the endoplasmic reticulum, and it is required in the exportation of proteins to the host cell by degrading the conserved motif Plasmodium export element (PEXEL), an indication that this enzyme is an important therapeutic target. Earlier research on the inhibition of *Pf*PMV was centred on the synthesis of different compounds that substantially look like the PEXEL but cannot be cleaved by PMV [33]. The idea was that the synthesized compound(s) would not be cleaved by *Pf*PMV; thus, no parasitic proteins would be exported to the host cell and ultimately prevent the survival of the malaria parasite. However, Ji et al. [32] reported that this type of inhibitor exhibited feeble inhibition of parasite growth, possibly due to its low membrane permeation ability or easy degradation by other enzymes. Similarly, Klemba and Goldberg [34] and Boddey et al. [35] in their studies were unsuccessful in attempts to disrupt the gene coding for the synthesis of PMV from both *P. falciparum* and *P. berghei.* As a result, the focus of this investigation was centred on identifying new scaffolds of nonpeptidomimetic drug candidate inhibitors. The screening of a library of small molecules that can inhibit aspartic proteases was carried out and led to the identification of a novel class of antimalarial compounds. Interestingly, attempts are being made to discover a safe and effective antimalarial drug from the range of compounds in this series.

According to reports, kaempferol is a natural flavonol found in plants and plant-based foods that is a derivative of flavonoids. It possesses potent antioxidant qualities that can hinder oxidative stress caused by the production of reactive oxygen species (ROS) [36, 37] occurring during malaria infections. In addition, malarial infections activate monocytes and neutrophils that generate ROS and oxidative stress [38] causing the degradation of hemoglobin [39, 40], thus signifying a correlation between antioxidant activity and antimalarial activity of kaempferol [41]. From the result obtained in this study, kaempferol inhibited *Pf*PMV in a noncompetitive manner, suggesting that kaempferol may bind to the regulatory site of plasmepsin V, to induce a conformational change, thereby preventing the exportation of several proteins necessary for the survival of *P. falciparum* in the host erythrocyte. The inhibition of this enzyme by kaempferol may be due to the antioxidative properties of kaempferol [37].

Quercetin has been detected in several fruits, vegetables, and medicinal plants such as Ginkgo biloba, *Hypericum perforatum*, *Allium cepa*, *Sambucus canadensis*, *Aesculus indica*, and *Dendrobium officinale* [42, 43]. Quercetin is known for its diverse biological and pharmacological properties such as anti-inflammatory [44], antioxidant [45], anticancer [46], and anti-HIV integrase activities [47] as well as having good effect against cardiovascular and neurodegenerative diseases [48]. Considering the effects of all the compounds screened, quercetin inhibited *Pf*PMV in a noncompetitive manner like kaempferol suggesting its binding at other sites apart from the active site of *Pf*PMV, ultimately hindering its activity. The result agreed with the finding of Ganesh et al. [49] who reported that quercetin exhibited antimalarial activity by inhibiting the growth of *P. falciparum* K1 and 3D7 strains. The inhibition of *Pf*PMV by quercetin may be suggested to be due to the antioxidative potentials of quercetin being a good antioxidant. Besides, most antioxidants have been reported to have good antimalarial effects [50, 51].

Shikonin is a type of naphthoquinone that is derived from traditional Chinese medicine. *Lithospermum* is demonstrated to have anti-inflammatory, antioxidant, anticancer, wound healing, and antimicrobial properties [52, 53]. The results of this study showed that shikonin displayed a competitive mode of inhibition against *Pf*PMV, an indication that it (shikonin) did not only compete with the substrate at binding at the active site of the enzyme but may mimic Plasmodium export element (PEXEL), thereby preventing the cleavage of PEXEL. Notwithstanding the inhibitory role of shikonin against *Pf*PMV, reports of antimalarial action of a related or derived naphthoquinone, 4-amino naphthoquinone, on *Plasmodium falciparum* have been established [54].

The discovery exhibited in the study with kaempferol, quercetin, and shikonin revealing the potential to inhibit *Plasmodium falciparum* plasmepsin V suggests important implications for malaria treatment. Plasmepsin V is a critical enzyme that is involved in the survival and proliferation of the malaria parasite, making it an attractive target for antimalarial drug development. The current treatment options for malaria, such as artemisinin-based combination therapies (ACTs), target different stages of the parasite's life cycle. However, the emergence of drug-resistant strains of the parasite highlights the need for novel antimalarial drugs with different modes of action. Hence, the identification of kaempferol, quercetin, and shikonin as inhibitors of plasmepsin V provides a potential new avenue for antimalarial drug development.

Molecular docking allows for the evaluation of a molecule's geometric fitness and affinity upon binding at the active site of a receptor [55]. It illustrates the ligand's affinity for the enzyme depicted with a negative score [23]. The most negative score of the ligand-enzyme complex is an indication of the better pose and affinity of the compound for the protein [56]. The higher negative values of kaempferol (-8.7 kcal/mol), shikonin (-8.2 kcal/mol), and quercetin (-8.1 kcal/mol) compared to other compounds are suggestive of their better interaction with *Pf*PMV; particularly, kaempferol revealing superior affinity for *Pf*PMV better than pepstatin, the standard (-8.3 kcal/mol), thus, might lend credence to its better efficacy and the structure-activity-based strategy used in this study. However, since molecular docking only evaluates a molecule's pose and affinity for the receptor, the top three compounds with the highest negative docking scores and interactions were taken further through molecular dynamic (MD) simulation. Interestingly, the higher docking scores reflected by the top three compounds were corroborated by binding energy results, particularly quercetin for *Pf*PMV in comparison to the reference standard and the other two compounds, indicative of greater binding efficiency and affinity of quercetin for this protein. Though, quercetin marginally had the lowest score among the compounds based on the docking scores, exposing the lack of predictive accuracy of molecular docking. Hence, the importance of MD simulation as a better or further confirmatory approach towards identifying potential drug candidates for drug development [57].

Molecular dynamic simulation is viable *in silico* tool that provides insight into how (dynamic) data at spatial atomic resolution can be obtained [58]. Additionally, it is a tool that studies the possible biological activity impact that may arise from complex instability and conformational alteration of the enzyme because of ligand binding [59]. Hence, it is germane to study the behaviour of the compounds at the binding pocket of the enzyme to understand the stability, compactness, and flexibility of the (ligand-enzyme) complex [60]. The RMSD quantifies the thermodynamic conformational stability of a protein-ligand complex during the MDS period; a lower RMSD indicates greater stability [57]. The fact that quercetin had a reduced RMSD value than the unbound *Pf*PMV indicates its ability to promote increased structural stability of *Pf*PMV; similarly, its lower RMSD value (*p* < 0.05) compared to pepstatin indicates its relative superiority as a *Pf*PMV inhibitor over the latter. This finding is consistent with the binding energy component profile, which showed quercetin to have the highest binding affinity for *Pf*PMV, implying that the resulting complex has greater structural stability and affinity. Above all, the top three compounds in this study had average RMSD values less than the acceptable limit of <3.5 Å, comparable to the value obtained with the unbound protein, indicating the potential and general stability of the compounds with *Pf*PMV. This is because Rosenberg [61] established that RMSD above >3.5 Å may be an indication of complex instability.

Another post-MD simulation metric is the RoG, which measures the total compactness of a complex; the higher the RoG value, the less complex the folding with conformational entropy [23]. While the RoG result obtained in this study is consistent with the RMSD result and energy component profile, the marginally lower RoG values of all the compounds and pepstatin relative to the unbound *Pf*PMV might only suggest the compactness of the complexes formed and the lowest values of quercetin and shikonin compared to pepstatin indicative of their stronger compactness (with *Pf*PMV) and better complex stability. Thus, it is implied that quercetin may be a potential lead and novel *Pf*PMV inhibitor. Overall, the findings of the current study on binding affinity and stability may demonstrate the potential affinity of the three compounds for *Pf*PMV, with quercetin having a better advantage as a probable lead compound.

The RMSF measures how the amino acid residues of a receptor move or fluctuate as a result of drug binding [23, 62]. An increased RMSF value indicates heightened flexibility of alpha-carbon atoms and unstable bonds [63]. The observed higher RSMF values of kaempferol and shikonin could only indicate that the two ligands possibly brought about an increase in their amino acid residue flexibility of *Pf*PMV complimenting their low binding affinities. The complexes of quercetin-*Pf*PMV and pepstatin-*Pf*PMV on the other hand witnessed reduced average RMSF values, suggesting a lessen flexibility of movement of the active site amino acid residues of *Pf*PMV. The lowest RMSF value of quercetin indicates a greater ability or rigidity to form stable bonds with *Pf*PMV protein. The RMSF result is entirely consistent with the energy component profile, RMSD and RoG findings of this study, which show quercetin to be the best in terms of potential affinity for *Pf*PMV and far superior to the effect shown by pepstatin. Thus, it is more stable and less distorted compared to kaempferol and shikonin.

The SASA plot measures the protein structure's exposure to the hydrophobic (solvent) environment [64]; the lower the SASA value, the more exposed the proteins' hydrophobic amino acid residues are, and the system's stability increase [65]. The binding of the studied three compounds resulted in the reduction of the SASA values when compared to unbound, *Pf*PMV, and reference compound, pepstatin, as witnessed in this study; this observation implies that more of the unbound and reference compound residues are sticking out to the solvent, indicating less stability. It is therefore evident that the three compounds bind favourably with *Pf*PMV, did not alter the exposure of the buried hydrophobic residues of *Pf*PMV, and ultimately did not adversely impact the systems' stability. Thus, this observation agrees with those of RMSD, RoG, and RMSF following the binding of kaempferol, quercetin, and shikonin and a further attestation that the structural integrity, which is significant for the inhibitory activity of *Pf*PMV, was not compromised.

The post-MD simulation interaction of a ligand (e.g., pepstatin, kaempferol, quercetin, and shikonin) against the active site amino acid residues of protein (*Pf*PMV) is usually attributable to the free binding energy of a complex [66]. The number and type or nature of interaction are a consequence of the resulting affinity [67]. The highest number of interactions (number of bonds) observed for pepstatin (20) was noticeable compared to quercetin with 16 bonds; this result was in tandem with thermodynamic energy components because the negative binding energy score of quercetin was marginally higher (*p* > 0.05) than pepstatin. The equal number of interactions between quercetin and shikonin is not corroborated or reflected in thermodynamic energy profiles with a reduced negative energy value of the former, indicating that some of the important energy bonds (such as conventional H bonds and van der Waals forces) in quercetin may not be contributing to the stability of the complex. Binding energy reflects all intermolecular forces or interactions between the ligand and protein and the extent of binding taking place [68].

In general, the current study's findings on binding affinity and stability demonstrated the potential affinity of the three compounds for *Pf*PMV, with quercetin having a better advantage as the lead compound. Although *in vitro* studies identified kaempferol as the best inhibitor of *Pf*PMV, MD simulation provides a better insight into the associated action mechanisms of the compounds on the protein folding and conformational changes.

## 5. Conclusion

The screening of the small molecular weight library of compounds avails the opportunity for the identification of new lead compounds with potential inhibitory effects on plasmepsin V. The novelty of this study resides in the effectiveness of the newly identified inhibitors in halting the hemoglobin degradation required for the survival of the malaria parasite. Hence, it can be suggested that by inhibiting plasmepsin V, these compounds might prevent parasitic modulation of host erythrocytes, leading to the clearance of malaria parasites in the host erythrocytes. Furthermore, computational studies revealed that the top three compounds investigated have a higher affinity, flexibility, compactness, and stability towards *Pf*PMV. Although *in vitro* studies showed kaempferol as the best compound, however, results from *in silico* studies established otherwise presenting quercetin as the most probable candidate for managing *P. falciparum*. Following the findings of this study, the three compounds could be further developed and evaluated as new *Pf*PMV *in vivo*. We, therefore, propose three new lead compounds, quercetin kaempferol, and shikonin, as novel aspartic protease inhibitors worthy of further investigation for malaria treatment.

## Figures and Tables

**Table 1:** IC_50_ values and kinetics of inhibition parameters for plasmepsin V inhibition by the screened compounds.

Compounds	IC_50_ (*μ*M)	Km (*μ*M)		Vmax (*μ*mol/min)	
		With	Without	With	Without
Pepstatin	62.60	ND	ND	ND	ND
Kaempferol	22.40	0.0820	0.0820	0.411	1.099
Quercetin	53.14	0.0790	0.0790	2.067	1.431
Shikonin	43.34	0.0730	0.6180	1.262	1.262

IC_50_: half-maximal inhibitory concentration; Km: concentration by which half of the enzyme active site is occupied by the substrate; Vmax: maximum reaction velocity at which all the enzyme becomes saturated with the substrate; ND: not determined.

**Table 2:** Docking scores of the six compounds and standard against *Pf*PMV.

S/N	Ligands	Docking scores (kcal/mol)
1	Pepstatin^∗^	-8.3
2	Kaempferol	-8.7
3	Shikonin	-8.2
4	Quercetin	-8.1
5	Doramectin	-7.2
6	5-Hydroxyl naphthoquinone	-6.7
7	Escin	-5.3

^∗^Reference standard.

**Table 3:** Energy component profiles of the reference standard and three compounds against *Pf*PMV.

Energy components (kcal/mol)					
Complexes	Δ*E*_vdW_	Δ*E*_elec_	Δ*G*_gas_	Δ*G*_solv_	Δ*G*_bind_
Pepstatin+*Pf*PMV	−46.45 ± 7.46	−170.71 ± 30.41	−217.16 ± 6.35	181.44 ± 27.92	−35.72 ± 9.82^a^
Kaempferol+*Pf*PMV	−27.46 ± 2.88	−17.70 ± 7.97	−45.17 ± 7.73	21.73 ± 4.65	−23.44 ± 4.28^b^
Quercetin+*Pf*PMV	−30.68 ± 3.05	−33.99 ± 4.84	−64.66 ± 4.28	28.10 ± 3.09	−36.56 ± 3.01^a^
Shikonin+*Pf*PMV	−31.59 ± 2.53	−8.80 ± 3.72	−40.39 ± 3.91	17.89 ± 2.53	−22.50 ± 2.76^b^

*Pf*PMV = *Plasmodium falciparum* plasmepsin V; Δ*E*_vdW_ = van der Waals energy; Δ*G*_bind_ = total binding free energy; Δ*G*_gas_ = gas phase free energy; Δ*E*_elec_ = electrostatic energy; Δ*G*_sol_ = solvation free energy. Values with different superscript letters are significantly different from each other at *p* < 0.05.

**Table 4:** RMSD, RoG, RMSF, and SASA values following complexation of top three compounds and pepstatin with *Pf*PMV.

Target	Ligands	RMSD (Å)	RoG (Å)	RMSF (Å)	SASA (Å)
*P*fPMV	Unbound	1.92 ± 0.27^a^	23.59 ± 0.11^a^	1.28 ± 0.59^a^	12323.14 ± 264.09^a^
	Pepstatin	2.01 ± 0.31^b^	23.53 ± 0.13^a^	1.23 ± 0.59^b^	12518.79 ± 349.16^b^
	Kaempferol	2.16 ± 0.26^b^	23.44 ± 0.13^a^	1.29 ± 0.59^a^	12297.89 ± 332.62^c^
	Quercetin	1.79 ± 0.15^c^	23.27 ± 0.09^a^	1.09 ± 0.47^c^	12275.66 ± 266.61^c^
	Shikonin	2.05 ± 0.26^b^	23.27 ± 0.09^a^	1.27 ± 0.69^a^	12190.77 ± 331.96^c^

*P*fPMV: *Plasmodium falciparum* plasmepsin V; RMSD: root mean square deviation; ROG: radius of gyration; SASA: solvent-accessible surface area; RMSF: root mean square fluctuation. Values with different superscript letters are significantly different from each other at *p* < 0.05.

## Data Availability

The data presented in this study are available in the article.

## References

[1] Nadjm B., Behrens R. H. (2012). Malaria:: an update for physicians. *Infectious Disease Clinics*.

[2] Escalante A. A., Cepeda A. S., Andreína Pacheco M. (2022). Why Plasmodium vivax and *Plasmodium falciparum* are so different? A tale of two clades and their species diversities. *Malaria Journal*.

[3] Bylicka-Szczepanowska E., Korzeniewski K. (2022). Asymptomatic malaria infections in the time of COVID-19 pandemic: experience from the Central African Republic. *International Journal of Environmental Research and Public Health*.

[4] Abubakr M., Sami H., Mahdi I. (2022). The phylodynamic and spread of the invasive Asian malaria vectors, Anopheles stephensi, in Sudan. *Biology*.

[5] WHO (2021). *World Malaria Report*.

[6] King P. (2021). *Insecticide Resistance and KDR Mutations in Malaria Vector from Different Transmission Sites in Uganda, [Ph.D. thesis]*.

[7] Abdishu M., Gobena T., Damena M., Abdi H., Birhanu A. (2022). Determinants of malaria morbidity among school-aged children living in East Hararghe Zone, Oromia, Ethiopia: a community-based case–control study. *Pediatric Health, Medicine and Therapeutics*.

[8] Al-Awadhi M., Ahmad S., Iqbal J. (2021). Current status and the epidemiology of malaria in the Middle East region and beyond. *Microorganisms*.

[9] UNICEF Childhood Diseases (Malaria, Pneumonia, Diarrhoea, HIV and Tuberculosis are preventable and treatable. But they are still killing children in large numbers). https://www.unicef.org/health/childhood-diseases.

[10] De Vrieze J. (2019). First malaria vaccine rolled out in Africa-despite limited efficacy and nagging safety concerns. *Science*.

[11] Dondorp A. M., Smithuis F. M., Woodrow C., von Seidlein L. (2017). How to contain artemisinin- and multidrug-resistant falciparum malaria. *Trends in Parasitology*.

[12] Dondorp A. M., Nosten F., Yi P. (2009). Artemisinin resistance in *Plasmodium falciparum* malaria. *New England Journal of Medicine*.

[13] Barnes C. B. G., Dans M. G., Jonsdottir T. K., Crabb B. S., Gilson P. R. (2022). PfATP4 inhibitors in the medicines for malaria venture malaria box and pathogen box block the schizont-to-ring transition by inhibiting egress rather than invasion. *Microbiology*.

[14] Wichers J. S., van Gelder C., Fuchs G. (2021). Characterization of apicomplexan amino acid transporters (ApiATs) in the malaria parasite *Plasmodium falciparum*. *Msphere*.

[15] Counihan N. A., Modak J. K., de Koning-Ward T. F. (2021). How malaria parasites acquire nutrients from their host. *Frontiers in Cell and Developmental Biology*.

[16] Gilson P. R., Chisholm S. A., Crabb B. S., de Koning-Ward T. F. (2017). Host cell remodelling in malaria parasites: a new pool of potential drug targets. *International Journal for Parasitology*.

[17] Bullen H. E., Charnaud S. C., Kalanon M. (2012). Biosynthesis, Localization, and Macromolecular Arrangement of the *Plasmodium falciparum* Translocon of Exported Proteins (PTEX). *Journal of Biological Chemistry*.

[18] Jiang S., Prigge S. T., Wei L. (2001). New class of small non-peptidyl compounds blocks *Plasmodium falciparum* development *in vitro* by inhibiting plasmepsins. *Antimicrobial Agents and Chemotherapy*.

[19] Sambrook J. (2001). Plasmid and their usefulness in molecular cloning. *Molecular Cloning, A Laboratory Manual*.

[20] Boonyalai N., Sittikul P., Yuvaniyama J. (2015). *Plasmodium falciparum* Plasmepsin V (PfPMV): insights into recombinant expression, substrate specificity and active site structure. *Molecular and Biochemical Parasitology*.

[21] Vishwanatha K. S., Rao A. A., Singh S. A. (2009). Characterisation of acid protease expressed from *Aspergillus oryzae* MTCC 5341. *Food Chemistry*.

[22] Lineweaver H., Burk D. (1934). The determination of enzyme dissociation constants. *Journal of the American Chemical Society*.

[23] Sabiu S., Balogun F. O., Amoo S. O. (2021). Phenolics profiling of *Carpobrotus edulis* (L.) N.E.Br. and insights into molecular dynamics of their significance in type 2 diabetes therapy and its retinopathy complication. *Molecules*.

[24] Mubarak Ali D., MohamedSaalis J., Sathya R., Irfan N., Kim J. W. (2021). An evidence of microalgal peptides to target spike protein of COVID-19: in silico approach. *Microbial Pathogenesis*.

[25] Aribisala J. O., Sabiu S. (2022). Cheminformatics identification of phenolics as modulators of penicillin-binding protein 2a of *Staphylococcus aureus*: a structure–activity-relationship-based study. *Pharmaceutics*.

[26] Kehinde I., Ramharack P., Nlooto M., Gordon M. (2022). Molecular dynamic mechanism (s) of inhibition of bioactive antiviral phytochemical compounds targeting cytochrome P450 3A4 and P-glycoprotein. *Journal of Biomolecular Structure and Dynamics*.

[27] Ryckaert J. P., Ciccotti G., Berendsen H. J. (1977). Numerical integration of the cartesian equations of motion of a system with constraints: molecular dynamics of n-alkanes. *Journal of Computational Physics*.

[28] Roe D. R., Cheatham T. E. (2013). *PTRAJ and CPPTRAJ: software for processing and analysis of molecular dynamics trajectory data*. *Journal of Chemical Theory and Computation*.

[29] Seifert E. (2014). OriginPro 9.1: scientific data analysis and graphing software-software review. *Journal of Chemical Information and Modeling*.

[30] Ylilauri M., Pentikäinen O. T. (2013). MMGBSA as a tool to understand the binding affinities of filamin–peptide interactions. *Journal of Chemical Information and Modeling*.

[31] Boonyalai N., Collins C. R., Hackett F., Withers-Martinez C., Blackman M. J. (2018). Essentiality of *Plasmodium falciparum* plasmepsin V. *PLoS One*.

[32] Ji X., Wang Z., Chen Q. (2022). In silico and in vitro antimalarial screening and validation targeting *Plasmodium falciparum* plasmepsin V. *Molecules*.

[33] Cheuka P. M., Dziwornu G., Okombo J., Chibale K. (2020). Plasmepsin inhibitors in antimalarial drug discovery: medicinal chemistry and target validation (2000 to present). *Journal of Medicinal Chemistry*.

[34] Klemba M., Goldberg D. E. (2005). Characterization of plasmepsin V, a membrane-bound aspartic protease homolog in the endoplasmic reticulum of *Plasmodium falciparum*. *Molecular and Biochemical Parasitology*.

[35] Boddey J. A., Hodder A. N., Günther S. (2010). An aspartyl protease directs malaria effector proteins to the host cell. *Nature*.

[36] Calderon-Montano J. M., Burgos-Moron E., Perez-Guerrero C., Lopez-Lazaro M. (2011). A review on the dietary flavonoid kaempferol. *Mini-Reviews in Medicinal Chemistry*.

[37] Holland T. M., Agarwal P., Wang Y. (2020). Dietary flavonols and risk of Alzheimer dementia. *Neurology*.

[38] Wallqvist A., Fang X., Tewari S. G., Ye P., Reifman J. (2016). Metabolic host responses to malarial infection during the intraerythrocytic developmental cycle. *BMC Systems Biology*.

[39] Tripathy S., Roy S. (2015). Redox sensing and signaling by malaria parasite in vertebrate host. *Journal of Basic Microbiology*.

[40] Omoregie E. S., Pal A. (2016). Antiplasmodial, antioxidant and immunomodulatory activities of ethanol extract of *Vernonia amygdalina* del. leaf in Swiss mice. *Avicenna Journal of Phytomedicine*.

[41] Pan W. H., Xu X. Y., Shi N., Tsang S. W., Zhang H. J. (2018). Antimalarial activity of plant metabolites. *International Journal of Molecular Sciences*.

[42] Li Y., Yao J., Han C. (2016). Quercetin, inflammation and immunity. *Nutrients*.

[43] Zahoor M., Shafiq S., Ullah H., Sadiq A., Ullah F. (2018). Isolation of quercetin and mandelic acid from *Aesculus indica* fruit and their biological activities. *BMC Biochemistry*.

[44] Lee H. N., Shin S. A., Choo G. S. (2018). Anti-inflammatory effect of quercetin and galangin in LPS-stimulated RAW264.7 macrophages and DNCB-induced atopic dermatitis animal models. *International Journal of Molecular Medicine*.

[45] Xu D., Hu M. J., Wang Y. Q., Cui Y. L. (2019). Antioxidant activities of quercetin and its complexes for medicinal application. *Molecules*.

[46] Nguyen L. T., Lee Y. H., Sharma A. R. (2017). Quercetin induces apoptosis and cell cycle arrest in triple-negative breast cancer cells through modulation of Foxo3a activity. *The Korean Journal of Physiology & Pharmacology*.

[47] Chaniad P., Wattanapiromsakul C., Pianwanit S., Tewtrakul S. (2016). Inhibitors of HIV-1 integrase from *Dioscorea bulbifera*. *Songklanakarin Journal of Science & Technology*.

[48] Salis C., Papageorgiou L., Papakonstantinou E., Hagidimitriou M., Vlachakis D. (2020). Olive oil polyphenols in neurodegenerative pathologies. *GeNeDis 2018: Genetics and Neurodegeneration*.

[49] Ganesh D., Fuehrer H. P., Starzengrüber P. (2012). Antiplasmodial activity of flavonol quercetin and its analogues in *Plasmodium falciparum*: evidence from clinical isolates in Bangladesh and standardized parasite clones. *Parasitology Research*.

[50] Kumar Mishra S., Singh P., Rath S. K. (2013). Protective effect of quercetin on chloroquine-induced oxidative stress and hepatotoxicity in mice. *Malaria Research and Treatment*.

[51] Laryea M. K., Sheringham B. L. (2021). Antimalarial, antioxidant, and toxicological evaluation of extracts of *Celtis africana*, *Grosseria vignei*, *Physalis micrantha*, and *Stachytarpheta angustifolia*. *Biochemistry Research International*.

[52] Kourounakis A. P., Assimopoulou A. N., Papageorgiou V. P., Gavalas A., Kourounakis P. N. (2002). Alkannin and shikonin: effect on free radical processes and on inflammation - a preliminary pharmacochemical investigation. *Archiv der Pharmazie: An International Journal Pharmaceutical and Medicinal Chemistry*.

[53] Papageorgiou V. P., Assimopoulou A. N., Ballis A. C. (2008). Alkannins and shikonins: a new class of wound healing agents. *Current Medicinal Chemistry*.

[54] Kapadia G. J., Azuine M. A., Balasubramanian V., Sridhar R. (2001). Aminonaphthoquinones- a novel class of compounds with potent antimalarial activity against *Plasmodium falciparum*. *Pharmacological Research*.

[55] Chen J., Wu S., Zhang Q., Yin Z., Zhang L. (2020). *α*-Glucosidase inhibitory effect of anthocyanins from *Cinnamomum camphora* fruit: Inhibition kinetics and mechanistic insights through *in vitro* and *in silico* studies. *International Journal of Biological Macromolecules*.

[56] Aribisala J. O., Nkosi S., Idowu K. (2021). Astaxanthin-mediated bacterial lethality: evidence from oxidative stress contribution and molecular dynamics simulation. *Oxidative Medicine and Cellular Longevity*.

[57] Verma A. K., Ahmed S. F., Hossain M. S. (2022). Molecular docking and simulation studies of flavonoid compounds against PBP-2a of *methicillin‐resistantStaphylococcusaureus*. *Journal of Biomolecular Structure and Dynamics*.

[58] Kumar Y., Singh H., Patel C. N. (2020). In silico prediction of potential inhibitors for the main protease of SARS-CoV-2 using molecular docking and dynamics simulation based drug-repurposing. *Journal of Infection and Public Health*.

[59] Childers M. C., Daggett V. (2017). Insights from molecular dynamics simulations for computational protein design. *Molecular Systems Design & Engineering*.

[60] Rampadarath A., Balogun F. O., Pillay C., Sabiu S. (2022). Identification of flavonoid c-glycosides as promising antidiabetics targeting protein tyrosine phosphatase 1B. *Journal of Diabetes Research*.

[61] Rosenberg M. S. (1972). *Sequence alignment: methods, models, concepts and strategies*.

[62] Ogidigo J. O., Iwuchukwu E. A., Ibeji C. U., Okpalefe O., Soliman M. E. (2022). Natural phyto, compounds as possible noncovalent inhibitors against SARS-CoV2 protease: computational approach. *Journal of Biomolecular Structure and Dynamics*.

[63] Shode F. O., Idowu A. S. K., Uhomoibhi O. J., Sabiu S. (2022). Repurposing drugs and identification of inhibitors of integral proteins (spike protein and main protease) of SARS-CoV-2. *Journal of Biomolecular Structure and Dynamics*.

[64] Beg A., Khan F. I., Lobb K. A., Islam A., Ahmad F., Hassan M. I. (2019). High throughput screening, docking, and molecular dynamics studies to identify potential inhibitors of human calcium/calmodulin-dependent protein kinase IV. *Journal of Biomolecular Structure and Dynamics*.

[65] Durham E., Dorr B., Woetzel N., Staritzbichler R., Meiler J. (2009). Solvent accessible surface area approximations for rapid and accurate protein structure prediction. *Journal of Molecular Modeling*.

[66] Izadi H., Stewart K. M., Penlidis A. (2014). Role of contact electrification and electrostatic interactions in gecko adhesion. *Journal of the Royal Society Interface*.

[67] Vergara R., Romero-Romero S., Velázquez-López I. (2020). The interplay of protein–ligand and water-mediated interactions shape affinity and selectivity in the LAO binding protein. *The FEBS Journal*.

[68] Parate S., Rampogu S., Lee G., Hong J. C., Lee K. W. (2021). Exploring the binding interaction of Raf kinase inhibitory protein with the N-terminal of C-Raf through molecular docking and molecular dynamics simulation. *Frontiers in Molecular Biosciences*.

